# Data on the changes of the mussels׳ metabolic profile under different cold storage conditions

**DOI:** 10.1016/j.dib.2016.03.051

**Published:** 2016-03-19

**Authors:** Violetta Aru, Maria Barbara Pisano, Francesco Savorani, Søren Balling Engelsen, Sofia Cosentino, Flaminia Cesare Marincola

**Affiliations:** a Department of Chemical and Geological Sciences, University of Cagliari, S.S. 554 Bivio Sestu, 09042 Monserrato (CA), Italy; b Department of Public Health, Clinical and Molecular Medicine, University of Cagliari, S.S. 554 Bivio Sestu, 09042 Monserrato (CA), Italy; c Spectroscopy & Chemometrics, Department of Food Science, University of Copenhagen, Rolighedsvej 26, 1958 Frederiksberg C, Denmark; d Department of Applied Science and Technology (DISAT), Polytechnic University of Turin, Corso Duca degli Abruzzi 24, 10129 Torino (TO), Italy

**Keywords:** Metabolomics, NMR, Microbiology, Mussels, Storage

## Abstract

One of the main problems of seafood marketing is the ease with which fish and shellfish undergo deterioration after death. ^1^H NMR spectroscopy and microbiological analysis were applied to get in depth insight into the effects of cold storage (4 °C and 0 °C) on the spoilage of the mussel *Mytilus galloprovincialis*. This data article provides information on the average distribution of the microbial loads in mussels׳ specimens and on the acquisition, processing, and multivariate analysis of the ^1^H NMR spectra from the hydrosoluble phase of stored mussels. This data article is referred to the research article entitled “Metabolomics analysis of shucked mussels’ freshness” (Aru et al., 2016) [1].

**Specifications Table**

**Table:** 

Subject area	*Chemistry, microbiology*
More specific subject area	*NMR-based Metabolomics*
Type of data	*Tables; Figures*
How data was acquired	^1^*H NMR (Varian Unity Inova 500 spectrometer, Agilent Technologies, CA, USA);*
*MestReNova (Version 8.1, Mestrelab Research SL);*
*SIMCA 13 software package (Umetrics, Umeå, Sweden)*
Data format	*Analyzed data*
Experimental factors	*Mussels were stored at different cold temperatures* (0 °*C and* 4 °*C*) *and sampled at different storage days*
Experimental features	*Integrated*^1^*H NMR-based metabolomics and microbiological analysis*
Data source location	*Cagliari, Sardinia (Italy)*
Data accessibility	*Data available within this article*

**Value of the data**

• The multivariate analysis of the NMR data highlights significant differences between the metabolic profile of fresh mussels (*Mytilus galloprovincialis*) and those stored at 4 °C and 0 °C.
• The microbiological analysis provides indication of the average distribution of the microbial species isolated from mussels׳ samples.
• These data would serve as an important reference for developing targeted analysis for applications in food research.

## 1 Data

Dataset provided in this article represents the results of a combined microbiological and NMR-based metabolomics investigation of mussels under different cold storage conditions (0 °C, 4 °C and different storage days). With respect to the analysis reported in the published manuscript [1], here we provide additional information on the mussels׳ microbiological characterization and on the Principal Component Analysis (PCA) and Orthogonal Partial Least Squares Discriminant Analysis (OPLS-DA) performed.

## 2 Experimental design, materials and methods

### 2.1 Sample collection and experimental design

**Fig. 1:**
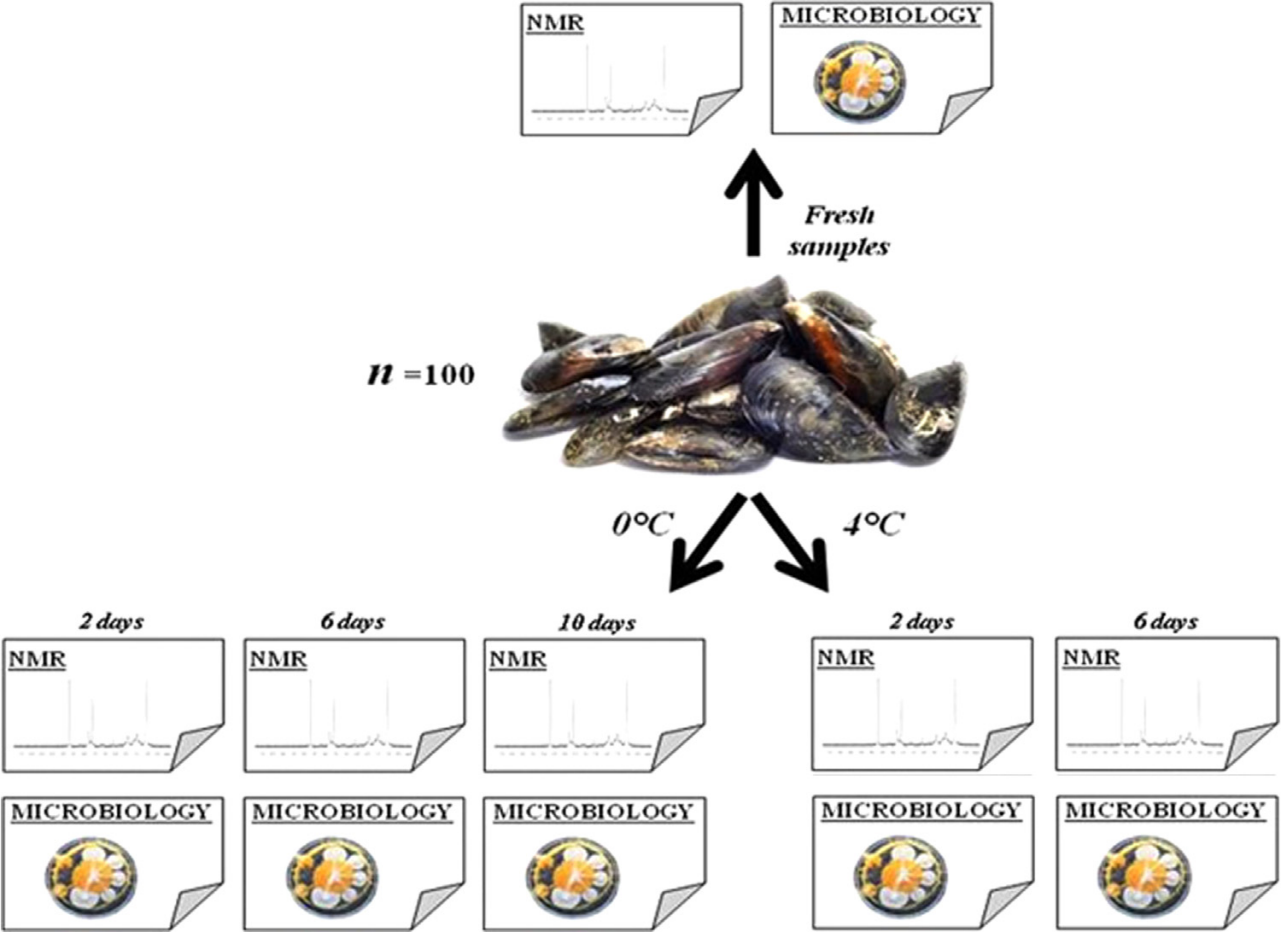
Experimental design for microbiological and NMR analysis on fresh and cold stored mussels.

Fresh live mussels (*Mytilus galloprovincialis*) were bought from a local seafood market in Cagliari (Italy) and immediately transported to the laboratory in portable coolers at approximately 4 °C. Mussels were subsequently inspected and dead ones or those with broken shells were discarded. The remaining mussels (100 individuals) were manually shucked with a sterile knife and each sample was put into insulated sterile plastic boxes without ice or water. Mussels were stored at 4 °C and 0 °C for 6 and 10 days, respectively. NMR and microbiological analysis were performed on fresh mussels (0 day) and after 2 and 6 days of storage at 4 °C and after 2, 6, and 10 days of storage at 0 °C (Fig. 1).

### 2.2 Microbiological analysis

**Fig. 2:**
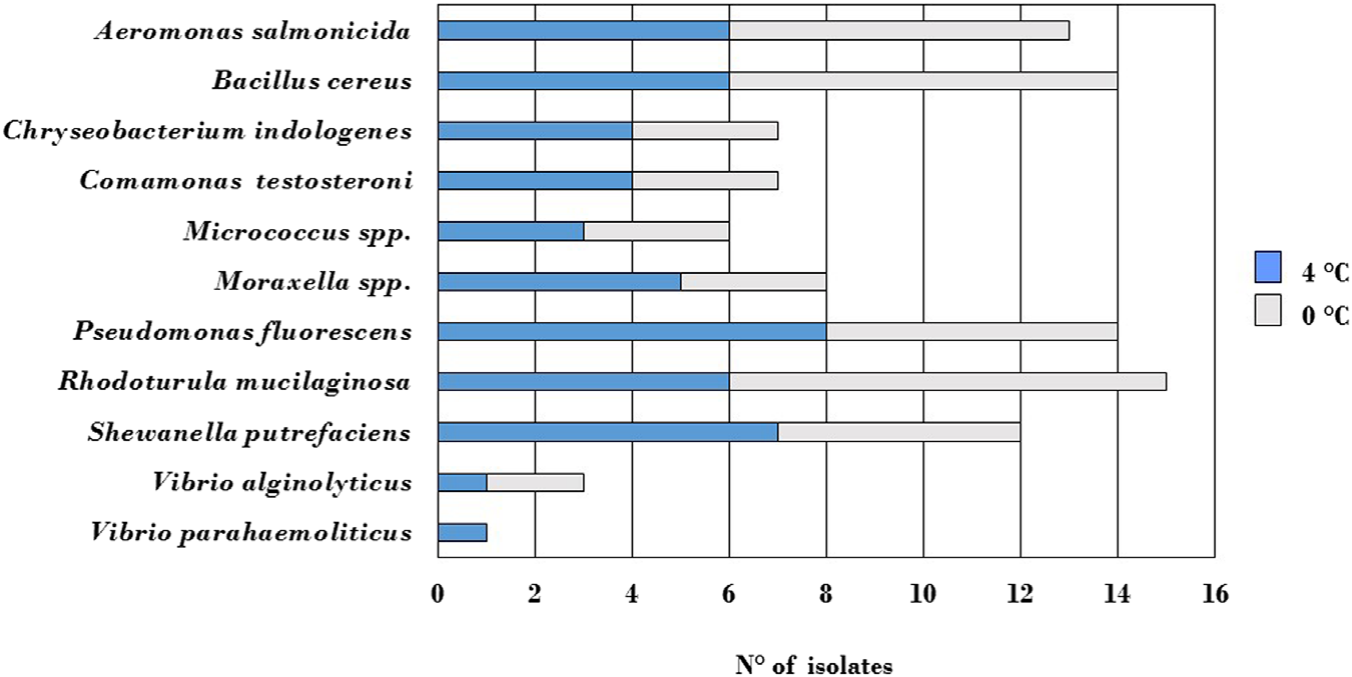
Average distribution of the microbial species isolated from mussels’ samples (*Mytilus galloprovincialis*) stored at 4 °C and 0 °C.

Microbiological analysis was performed as previously described [1]. Total Counts of Mesophilic (MMC) and Psychrotrophic (PMC) microorganisms were determined by the pour plate method, using Plate Count Agar (PCA, Microbiol, Cagliari, Italy) and incubating at 30 °C for 48 h and at 4 °C for 7 days, respectively. Two-way ANOVA was performed on microbiological data, with temperature and time as factors, using GraphPad Prism Statistics software package version 3.00 (GraphPad Prism Software Inc., San Diego, CA, USA). Statistical significance was inferred at *p*<0.01. The average distribution of the microbial species isolates from mussels’ samples is shown in Fig. 2.

### 2.3 Sample preparation and NMR analysis

Water-soluble metabolites were extracted according to the Folch method [2]. ^1^H-NMR experiments were performed at 300 K on a Varian Unity Inova 500 spectrometer (Agilent Technologies, CA, USA) operating at the frequency of 499.84 MHz. One-dimensional (1D) ^1^H NMR spectra were obtained by applying a presaturation technique with low power radiofrequency irradiation for 1.5 s to suppress solvent (water) residual signal. For each spectrum, a total of 256 scans were collected into 64k points over a spectral width of 6000 Hz, with a 45° pulse, an acquisition time of 1.5 s, and a relaxation delay of 4 s. After Fourier transformation with 0.3 Hz line broadening, spectra were phased and baseline corrected, and the chemical shift scale was set by assigning a value of δ=0.00 ppm to the signal for the internal standard sodium 3-trimethylsilyl-propionate-2,2,3,3,-d4 (TSP). NMR spectra displayed several hundred peaks that arise from the different functional groups of a large number of metabolites including amino acids, organic acids, organic osmolytes, and carbohydrates. A total of 29 metabolites were identified whose assignments, multiplicity and chemical shifts are reported in Table 1.

### 2.4 Chemometrics analysis

NMR spectra were manually phased and baseline corrected using MestReNova (Version 8.1, Mestrelab Research SL). Each NMR spectrum was integrated between 0.5 and 9.5 ppm over a series of 0.005 ppm integral regions (bins). The regions between 4.6 and 5.0 ppm and 0.5 and 0.5 ppm were excluded because of the signals of water and TSP, respectively. The noisy region between 9.5 and 10.5 ppm was also removed. NMR data set, sized 62 samples and 1665 variables, was converted into an Excel file and then imported to SIMCA version 13.0 (Umetrics, Umeå, Sweden) for statistical analysis.

**Fig. 3:**
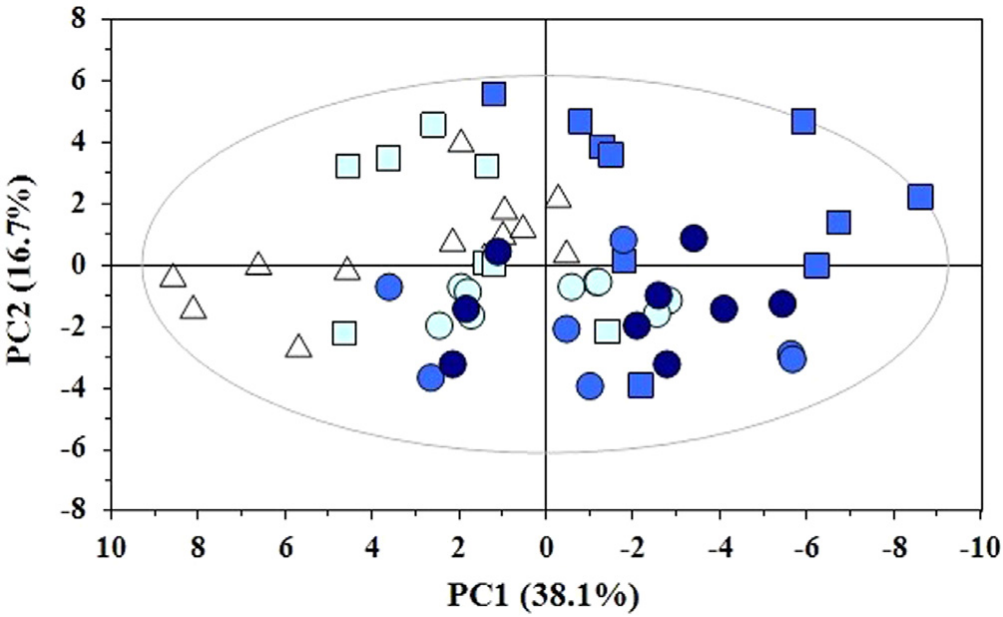
PCA scores plot of NMR spectra of mussels water-soluble extracts (*R*^2^*X*=0.878, *Q*^2^=0.679). Scores symbol denotes the storage temperature (Δ, fresh samples; ●, 0 °C; ■, 4 °C), while color indicates the storage days (light blue, 2d; blue, 6d; dark blue, 10d).

Principal components analysis (PCA) [3] of the ^1^H NMR spectra of the mussels׳ water-soluble extracts was performed to sample overview and to show trends, groupings and outliers in the data (Fig. 3).

Orthogonal Partial Least Squares Discriminant Analysis (OPLS-DA) [4] was applied at each temperature, in pairwise comparisons, to extract and maximize the systematic differences between experimental groups and to focus on the metabolic variations at the different time points. Model validity was assessed by using permutation tests (Y-scrambling) and CV-ANOVA [5]. The performance parameters of the models are reported in Table 2.

The OPLS discriminant analysis allowed the identification of several putative biomarkers of mussels׳ freshness. The identified discriminant metabolites were quantified from the ^1^H-NMR spectra and their relative amount is reported in Table 3.

## Figures and Tables

**Table 1:** List of the metabolites identified in the ^1^H NMR spectra of mussels’ hydrosoluble extract.

**Reference**⁎	**Metabolites**	**Multiplicity**^a^	**ppm**
**1**	Isoleucine	*t*	0.93
		*d*	1.00
**1**	Leucine	*t*	0.96
**1**	Valine	*d*	0.98
		*d*	1.03
**2**	Unknown	*s*	1.10
**3**	Lactate	*d*	1.31
**4**	Alanine	*d*	1.48
**5**	Unknown	*d*	1.50
**6**	Arginine	*m*	1.65
		*m*	1.72
**7**	Acetate	*s*	1.91
**8**	Methionine	*m*	2.16
**9**	Acetoacetate	*s*	2.24
**10**	Glutamate	*dd*	2.35
**11**	Succinate	*s*	2.40
**12**	β-alanine	*t*	2.55
**13**	Hypotaurine	*t*	2.64
**14**	TMA	*s*	2.90
**15**	Betaine (s), Taurine (t), TMAO (s)	*–*	3.26
**16**	Taurine	*t*	3.41
**17**	Glycine	*s*	3.54
**18**	Betaine	*s*	3.91
**19**	Homarine	*s*	4.35
**20**	α-Glucose	*d*	5.24
**21**	Maltose	*d*	5.40
**22**	Glycogen	*bb*	5.41
**23**	Uracil	*d*	5.80
**24**	Inosine	*d*	6.11
**25**	Fumarate	*s*	6.50
**26**	Tyrosine	*d*	6.91
		*d*	7.20
**27**	Histidine	*s*	7.18
		*s*	8.08
**28**	Phenylalanine	*d*	7.31
		*m*	7.36
		*m*	7.42
**29**	Formate	*s*	8.45

a *s*: singlet, *d*: doublet, *t*: triplet, *dd*: doublet of doublets, *m*: multiplet, *bb*: broad band.

⁎ Peak references are taken from Fig. 1 in Ref. [1].

**Table 2:** Performance parameters of the OPLS-DA models.

		**OPLS-DA parameters**					
					*Permutation test* (*n*=200)		
**Storage temperature (°C)**	**Comparison Group (days*****vs*****days)**	*R*^*2*^*X*	*R*^*2*^*Y*	*Q*^*2*^*Y*	*R*^*2*^*Y* intercept	*Q*^*2*^*Y* intercept	a*p-value*
0	0 *v*s 2	0.875	0.988	0.833	0.294	−0.284	0.0047
2 *vs* 6/10	0.715	0.743	0.512	0.305	−0.185	0.011
4	0 *vs* 2	0.639	0.917	0.648	0.410	−0.2	0.017
2 *vs* 6	0.618	0.938	0.864	0.401	−0.294	0.00001

a *p* values were obtained from analysis of variance testing of cross-validated predictive residuals (CV-ANOVA).

**Table 3:** Relative amounts of discriminant metabolites. Values are reported as mean±SD.

				**Metabolites**									
***Storage temperature***		***Days of storage***		***Ace***	***Ala***	***BCAA***	***Bet***	***Glc***	***Hom***	***Lac***	***Suc***	***TMA***	***Tau***

		**0**		0.30±0.12	1.35±0.71	1.30±0.41	1.59±0.71	1.01±0.49	1.69±0.50	0.50±0.20	1.12±0.43	0.05±0.01	1.64±0.83
**4** °**C**		**2**		0.73±0.22⁎⁎	1.45±0.32⁎	1.37±0.28⁎⁎	1.59±0.30⁎	1.18±0.36⁎⁎	1.50±0.51	0.91±0.34⁎⁎	1.39±0.50⁎	0.14±0.06⁎⁎	1.32±0.57
**6**		5.40±0.82⁎⁎	2.11±0.74⁎	2.00±0.70⁎⁎	1.35±0.42	1.78±0.32⁎⁎	1.39±0.47	4.01±0.79⁎⁎	1.89±0.72⁎	7.29±0.90⁎⁎	1.24±0.22
**0** °**C**		**2**		0.35±0.15⁎⁎	1.37±0.66	1.30±0.43	1.49±0.41	1.12±0.29	1.48±0.40⁎⁎	0.91±0.30⁎	1.58±0.37⁎	0.14±0.03⁎⁎	1.49±0.30
**6**		0.53±0.15⁎⁎	1.38±0.38	1.38±0.35	1.39±0.48⁎	1.24±0.53	1.41±0.36⁎⁎	0.10±0.39⁎	1.99±0.75⁎	1.07±0.29⁎⁎	1.39±0.77
**10**		1.03±0.38⁎⁎	1.82±0.21	1.40±0.45	0.96±0.24⁎	1.27±0.36	1.00±0.38⁎⁎	1.13±0.79⁎	2.03±0.28⁎	1.20±0.47⁎⁎	0.99±0.27

Keys: Ace=acetate; Ala=alanine; BCAA=branched chain amino acids; Bet=betaine; Glc=glucose; Hom=homarine; Lac=lactate; Suc=succinate; TMA=trimethylamine; Tau=taurine.

⁎ *p*≤0.05.

⁎⁎ *p*≤0.01.
