# Supplementary material for: Data on the changes of the mussels׳ metabolic profile under different cold storage conditions
Source: Data Brief. 2016 Mar 19;7:951–7. doi: 10.1016/j.dib.2016.03.051 (PMC4818350; doi:10.1016/j.dib.2016.03.051)
Supplement: Supplementary file 1 — Supplementary material [file mmc1.pdf]

Conflict of Interest Statement

Manuscript title: *Data on the changes of the mussels' metabolic profile under different cold storage conditions*

The authors whose names are listed immediately below certify that they have NO affiliations with or involvement in any organization or entity with any financial interest (such as honoraria; educational grants; participation in speakers' bureaus; membership, employment, consultancies, stock ownership, or other equity interest; and expert testimony or patent-licensing arrangements), or non-financial interest (such as personal or professional relationships, affiliations, knowledge or beliefs) in the subject matter or materials discussed in this manuscript.

Flaminia Cesare Marincola
Violetta Aru
Maria Barbara Pisano
Francesco Savorani
Søren Balling Engelsen
Sofia Cosentino

This statement is signed by Flaminia Cesare Marincola, that in the role of the corresponding author acts as a representative of the other co-authors to indicate agreement that the above information is true and correct.

Sincerely
